# Multiferroic and Magnetodielectric Effects in Multiferroic Pr_2_FeAlO_6_ Double Perovskite

**DOI:** 10.3390/nano12173011

**Published:** 2022-08-30

**Authors:** Sheng Liu, Feng Xiang, Yulan Cheng, Yajun Luo, Jing Sun

**Affiliations:** 1 Hunan Institute of Engineering, College of Mechanical Engineering, No.88, East Fuxing Road, Xiangtan 411104, China; 2 Hunan Institute of Engineering, School of Electrical and Information Engineering, Xiangtan 411104, China

**Keywords:** double perovskites, multiferroics, Pr_2_FeAlO_6_, magnetodielectric response

## Abstract

Single-phase multiferroics that allow the coexistence of ferroelectric and magnetic ordering above room temperature are highly desirable, and offer a fundamental platform for novel functionality. In this work, a double perovskite multiferroic Pr_2_FeAlO_6_ ceramic is prepared using a sol-gel process followed by a quenching treatment. The well-crystallized and purified Pr_2_FeAlO_6_ in trigonal structure with space group R3c is confirmed. A combination of the ferroelectric (2P_r_ = 0.84 μC/cm^2^, E_c_ = 7.78 kV/cm at an applied electric field of 20 kV/cm) and magnetic (2M_r_ = 433 memu/g, H_c_ = 3.3 kOe at an applied magnetic field of 1.0 T) hysteresis loops reveals the room-temperature multiferroic properties. Further, the magnetoelectric effect is observed from the measurements of magnetically induced dielectric response and polarization. The present results suggest a new complex oxide candidate for room-temperature multiferroic applications.

## 1. Introduction

Single-phase multiferroic compounds that allow the coexistence of ferroelectric and magnetic ordering have spurred interest not only in fundamental physics, but also in potential applications for actuators, energy harvesting devices, spintronics, and memory elements [1,2,3,4]. However, naturally occurring and artificially synthesized single-phase multiferroics exhibit a virtually weak magnetoelectric effect below room temperature [5,6,7], hindering their practical applications. A_2_BB′O_6_ (A, rare earth and B/B′, transition metals) ordered double perovskites (DPs) are a type of promising multiferroics, due to their remarkable magnetoresistive, electronic properties, high Curie temperature, and high degree of spin polarization [8,9,10]. DPs are built up by the doubling of a unit cell of prototypical perovskite structure (ABO_3_), where the BO_6_ and B′O_6_ octahedra are alternatively arranged in two interleaving sublattices. Depending on the combinations of A and B/B′ sites, DPs provide rich configurations for engineering coexistence of ferromagnetism and ferroelectricity. Researchers have theoretically and experimentally explored many rare earth element-resolved DPs to determine possible multiferroic compounds, such as La_2_NiMnO_6_ [11,12], Dy_2_FeCrO_6_ [13], Sm_2_NiMnO_6_ [14], and Pr_2_FeCrO_6_ [15]. Because of a high degree of symmetry, the readily formed antisite defects, and the difficult fully ordered arrangement of B/B′ sites in double perovskite structure, double perovskite shows simultaneously feeble ferromagnetism and ferroelectricity at room temperature.

In reality, the magnetic properties in A2BB′O6 perovskites can be controlled by tuning the interactions between the B and B′ ions in octahedral as well as A-site symmetries [16,17,18]. Meanwhile, the substitution of A-site by a rare earth element changes the B-O-B′ bond angles and exchange interactions, facilitating magnetism [19]. For a typical example, in the Pr_2_FeCrO_6_ compound, its symmetry controls the magnetic property as it possesses room-temperature ferromagnetism ordering with R3c symmetry [20] and paramagnetic ordering with Pbnm symmetry [21]. In a non-symmetrical structure, the net spin contribution stems from B–B′ interactions with rare earth ions, leading to ferromagnetic ordering. S. Ravi theoretically demonstrated that Pr_2_FeCrO_6_ had a higher Curie temperature (Tc) in R3c symmetry than that in Pbnm [22]. In addition, in R3c symmetry, the net spin contribution arose from Fe–Cr interactions with Pr ions, which did not take part in Pbnm symmetry. For electric polarization, polarizability can also be driven by the square bond formation in the B or B′ ions and the oxygen vacancies. One of the cases is the polarization enhancement in Y_2_FeAlO_6_ that is induced by introducing non-magnetic aluminum ions into the B-site [23].

The degree of the B-site ordering for perovskites depends on the synthesis method, temperature, and strain engineering, ranging from fully ordered (an ordered rock-salt type) to fully disordered type [24,25]. A surge in the cation ordering increases the magnetic ordering. Thermodynamically, an ordered sample with optimum magnetic properties is unable to be realized by the conventional solid-state reaction [15]. A quenching treatment is an efficacious route to create an oriented structure and to reduce antisite defects [26,27]. In addition, a high cooling rate associated with quenching leads to an increase in electrical polarization in perovskite, in favor of observing room-temperature polarization.

From the above viewpoints, in this work, we provide a new element-resolved double perovskite Pr_2_FeAlO_6_, which is prepared using a solid-state reaction followed by a quenching treatment. The magnetic, ferroelectric characteristics and magnetodielectric (MD) coupling are determined. An understanding of room-temperature multiferroic behavior could be effectively utilized for magnetically tailored electrical applications.

## 2. Materials and Methods

The polycrystalline Pr_2_FeAlO_6_ samples were prepared using a sol-gel process with a quenching treatment. Stoichiometric amounts of analytical reagent grade precursor Pr(NO_3_)_3_·6H_2_O (≥99.9%, Sigma Aldrich), Fe(NO_3_)_3_·9H_2_O (≥99.9%, Sigma Aldrich), and Al(NO_3_)_3_·9H_2_O (≥99.9%, Sigma Aldrich) were weighted and dissolved in a dilute HNO_3_ solution. Citric acid was used as a complex, and ethylene glycol was used as a solvent. To obtain the xerogel, the precursor was fully dissolved, evaporated under continuous mechanical stirring for 4 h, and dried at 120 °C. Afterwards, xerogel was ground, and thermally treated at 450 °C for 4 h in air. Subsequently, the sintered powder was ball-milled, dried, and granulated by sieving. The sintered powders together with 5% PVA (polyvinyl alcohol) were compressed into tablets under a uniaxial pressure of 60 MPa with the size of 10 mm in diameter and 2 mm in thickness. Finally, pressed pellets were sintered at 1050 ◦C for 1 h, removed from the furnace and then cooled in Ar atmosphere for 20 min. 

The crystalline phases were identified by X-ray diffraction (XRD, Philips X-pert PRO, PANalytical, Almelo, The Netherlands) with the Rietveld refinement method using GSAS. The morphology and microstructure were examined using a scanning electron microscope (SEM, SU3500-SE, Hitachi, Ltd., Tokyo, Japan). The phase formation analysis was characterized by using a transmission electronmicroscopy (TEM, FEI Tecnai-T12, USA). The room-temperature Fourier transform infrared spectroscopic (FTIR) studies were performed using a spectrometer (Spectrum BX, Perkin Elmer, Waltham, MA, USA). The magnetic measurements were carried out using a physical property measurement system (PPMS). The dielectric characteristics were performed by combining a HP4284 LCR meter (Agilent, Santa Clara, CA, USA), and the ferroelectric hysteresis loop was evaluated by using a ferroelectric tester based on the Sawyer Tower circuit (Precision Multiferroic II, Radiant, USA). The magnetic field source for the magnetodielectric and magnetically induced polarization measurements was a custom-designed magnet with a maximum value of 1.0 T, and the field direction was perpendicular to the rotation symmetry axis of the pellet.

## 3. Results and Discussions

**Figure 1:**
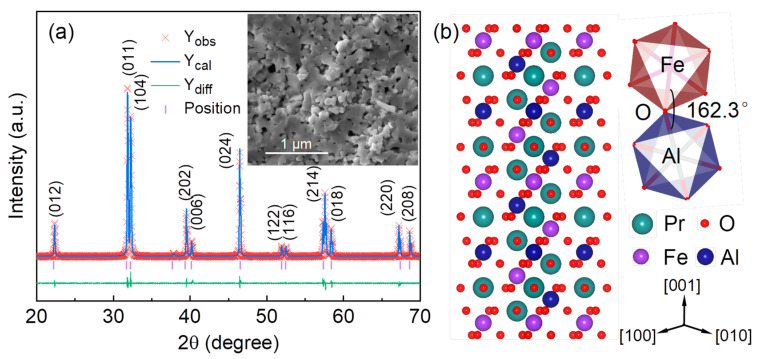
(**a**) X-ray diffraction pattern of Pr_2_FeAlO_6_ ceramic. Inset shows the SEM image. (**b**) The crystal structure of double perovskite Pr_2_FeAlO_6_. The crystallographic is constructed by the crystal structure visualization software (Diamond) and the lattice constants are obtained from the Rietveld structure refinement.

Figure 1a shows the XRD pattern of the as-synthesized ceramic. The pattern confirms the purification of Pr_2_FeAlO_6_, and all the diffraction peaks can be indexed with the R3c space group without any notable impurity phases. The crystal structure reported here is similar to the reported nanopolycrystalline Pr_2_FeCrO_6_ [20]. The lattice constants recorded using the Rietveld method for structure refinement (R factors *R_p_* (3.55%), *w_Rp_* (4.45%), and *χ*^2^ (2.39%)) are found to be a = b = 5.511 ± 0.026 Å, and c = 13.0674 ± 0.071 Å. The values of the atomic positions; the temperature factors; and the U, V, and W parameters obtained from the refinement are shown in Supplementary Table S1. The low symmetry in the R3c space group for Pr_2_FeAlO_6_ by a quenching treatment permits Fe/Al ordering on the B-site. The symmetry and stability of perovskite crystal are often understood in terms of the tolerance factor t, defined as t = (r_O_ + r_A_)/√2(r_O_ + r_B_), where r_O_, r_A_, and r_B_ are the O, A-site, and B-site effective radii, respectively [28]. The tolerance factors of 0.912 deviated largely from 1, indicating the presence of A-O or B-O bond length change or octahedral tilting. The microstructure of the Pr_2_FeAlO_6_ pellet is depicted in the inset of Figure 1a. Nano- and micrometer-scale mixed grains are observed. Similar particle morphology can also be observed in the Gd_2_FeCrO_6_ double perovskite synthesized using a sol-gel process [29]. The compositional semi-quantitative analysis confirms the stoichiometry of Pr, Fe, and Al in the ratio of 1.96:1:1.05. A perspective view of the crystal structure of Pr_2_FeAlO_6_ is depicted in the inset of Figure 1b. The octahedral Fe and Al are assembled within the distorted octahedral tilting in the antiphase orientation, arranged in a rock-salt ordered arrangement.

**Figure 2:**
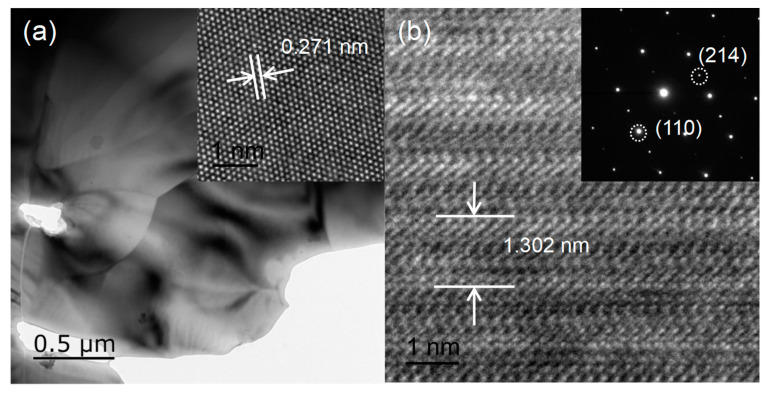
(**a**) TEM and (**b**) HRTEM image of Pr2FeAlO6. Inset of (**b**) shows SAED. The viewing direction is parallel to the <010> direction.

Formation of a well-crystallized compound was confirmed by a thorough TEM/HRTEM and SAED analysis, as shown in Figure 2. Sharp edges for the Pr2FeAlO6 evidence the high crystalline nature. The lattice fringe shown in the inset of Figure 2a indicates the (110) plane orientation corresponding to d-spacing of 0.271 nm. The lattice parameter c shown in Figure 2b along (001) orientation can be also determined to be 1.302 nm, which is consistent with the previous XRD result. The selected area diffraction pattern (SAED) with a regular and clear hexagonal diffraction confirms the polycrystalline nature of the Pr2FeAlO6 sample. The results obtained from HRTEM and SAED studies agree well with the XRD results, which further evidence the phase formation. 

**Figure 3:**
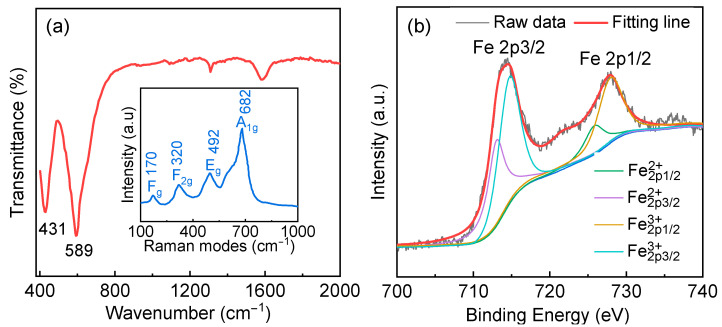
(**a**) Room-temperature FTIR spectrum and Raman spectra of the Pr_2_FeAlO_6_ ceramic sample; (**b**) X-ray photoemission experiment performed at room temperature for Pr_2_FeAlO_6_.

To examine the local structural distortion, a room temperature FTIR spectrum was recorded in the range from 400 to 1000 cm^−^^1^. The transmission mode 431 cm^−^^1^ is related to Fe/Al–O bending vibrations of Fe/AlO_6_ octahedral [30,31]. The broad transmission band observed around 589 cm^−^^1^ is correlated to the anti-symmetric stretching vibrations of Fe/Al–O bonds within octahedra [32,33]. A small shift of this mode compared to symmetry perovskite with Pbnm group space in Pr_2_FeAlO_6_ is attributed to the occurrence of octahedral lattice strain induced by rapid cooling treatment. The recorded room-temperature Raman spectrum of Pr_2_FeAlO_6_ is shown in inset of Figure 3a. The Raman spectrum is found to be dominated by three peaks at 320 cm^−^^1^, 492 cm^−^^1^, and 682 cm^−^^1^, which are assigned to the symmetric bending vibration, antisymmetric stretching and symmetric stretching vibrations of the octahedra, respectively [13,34]. Also, the low vibration mode located at 170 cm^−^^1^ may be related to the stretching vibrations of Pr ion, which is generated by the lattice translation [34]. Because of the non-centrosymmetric position of Pr ions, the ground-state energy is split. The interaction between rare-earth and iron can impact this translation. Such displacement gives evidence of magnetic interaction in sublattice. Figure 3b shows the 2p3/2 and 2p1/2 XPS spectra for Pr_2_FeAlO_6_ sample. Spin-orbit split 2p3/2 and 2p1/2 of Fe2p region appear at 714.2 and 727.1 eV, respectively. The observed asymmetric broad peak indicates the coexistence of Fe^3+^ and Fe^2+^. The relative amount of the Fe^3+^ to Fe^2+^ ion ratio has been quantitatively analyzed by the integration of peak areas for both ions. The peak ratio (the sum of the peak area ${\mathrm{Fe}}_{2p3/2}^{3+}$ and ${\mathrm{Fe}}_{2p1/2}^{3+}$ divided by the sum of peak area ${\mathrm{Fe}}_{2p3/2}^{2+}$ and ${\mathrm{Fe}}_{2p1/2}^{2+}$) of Fe^3+^/Fe^2+^ is 4.11. The partial conversion of Fe^3+^ to Fe^2+^ arises from the substitution of Fe ion by Al ion, and the total charge balance of the sample may be compensated by the oxygen vacancies. The presence of Fe^2+^ lowers the intensity of the high energy region and weakens the orbital hybridization intensity at A-site and oxygen, which elongates the bond length between Pr^3+^ and O^2^^−^ and leads to polarization enhancement [23].

**Figure 4:**
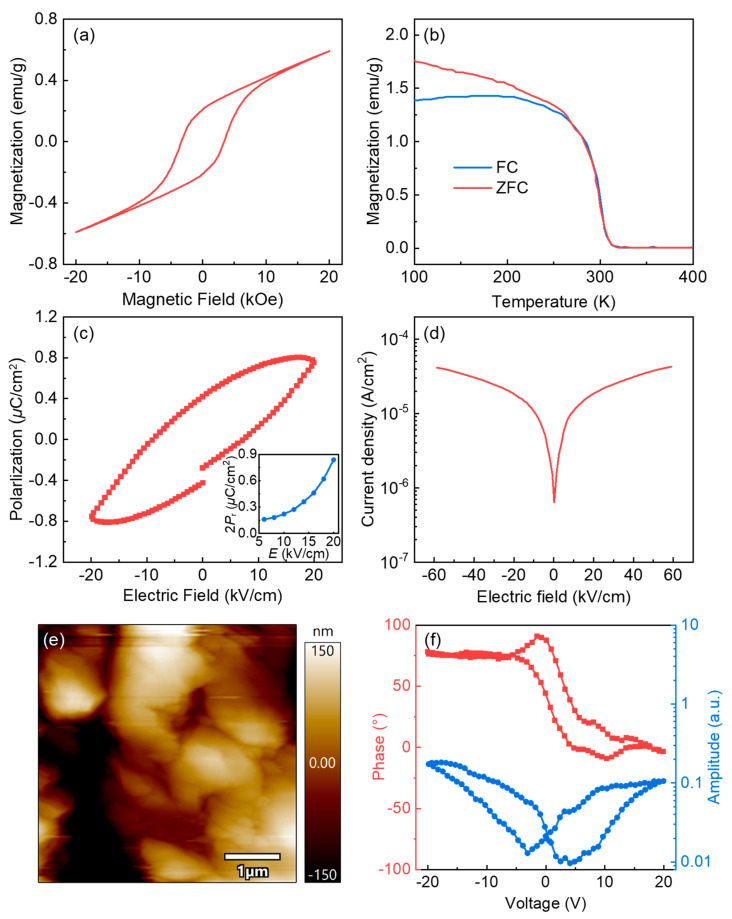
(**a**) Field-dependent and (**b**) temperature-dependent magnetization of Pr2FeAlO6 compound. (**c**) Ferroelectric hysteresis loops at room temperature for Pr2FeAlO6. Inset shows the values of remnant polarization 2Pr as a function of electric field.(**d**) The leakage current density. (**e**) Topography with corresponding PFM image. (**f**) The local butterfly-type piezoresponse hysteresis loop and polarization switching.

To evaluate the magnetic properties of Pr_2_FeAlO_6_, a temperature-dependent magnetization curve was considered at an applied magnetic field of 0.1 T. Evidently, magnetization sharply decreases with the lowering temperature and an extrapolating line intersects at 319 K (Curie temperature). The divergence of FC and ZFC at a temperature below Curie temperature may suggest a hysteresis process. The FC and ZFC magnetization values both increase when the temperature decreases from 319 K to 100 K. The change of ZFC protocol indicates that Pr_2_FeAlO_6_ has a typical FM behavior, suggesting that the superexchange of Fe^3+^-O-Al^3+^ plays a dominant role in this temperature range [35]. In the FC protocol, the magnetic moment tends to align along the direction of the applied magnetic field. An increase in temperature results in an increase in thermal energy. The thermal energy interferes with the alignment of the Fe and Al sublattice (B-site) magnetic moments, resulting in a significant decrease in the net magnetization. Pr_2_FeAlO_6_ possesses weak room-temperature ferromagnetism (FM), as displayed in Figure 4a. The saturation polarization (M_s_) is 0.59 emu/g, which is higher than that in multiferroicity Ba_2_FeMnO_6_ (0.122 emu/g) [36] and Y_2_FeAlO_6_ (0.359 emu/g) [23] ceramics. Das et al. reported that polycrystalline micron-sized grains in DPs unfavored the Fe^3+^–O^2−^–Fe^3+^ magnetic interaction [15]. In our case, the magnetic interaction in the Pr_2_FeAlO_6_ compound originates from the special magnetic moment arrangement of the Fe^3+^ ions and the exchange interaction of spin Pr ions with neighboring Fe ions. On the one hand, in the R3c space group, the inclination of the FeO_6_ octahedron strengthens the long-range ferromagnetic ordering and dwindling of anti-site defects. On the other hand, the incorporation of a smaller ion radius Al^3+^ increases the tilt angle of the FeO_6_ octahedron (seen in Figure 1b), thus, enhancing the ferromagnetism. Figure 4c shows polarization versus electric field (P-E) loop measured at room-temperature for Pr_2_FeAlO_6_ ceramic. Judging from the P-E loop and the recorded current density (Figure 4d), the sample is relatively leaky, therefore ferroelectric domain morphology of the sample using piezoresponse force microscopy (PFM) was carried out. Figure 4e shows the topography and the corresponding PFM image. The amplitude image reflects the strength of the piezoelectric signal in Pr_2_FeAlO_6_. Under the same applied electric field, the amplitude signal is different due to the different deformation of ferroelectric domains. When a direct current voltage of up to 20 V was applied, the sample exhibited a typical butterfly-type piezoresponse hysteresis loop (Figure 4f). The observation of domain signal and its switching behaviour confirm the occurrence of ferroelectricity in Pr_2_FeAlO_6_. In the P-E loop, the values of remnant polarization 2P_r_ and coercivity are 0.84 μC/cm^2^ and 3.89 kV/cm. In the inset, 2P_r_ increases with increasing the driving field. In the DPs, the polarization mechanism is related to the exchange-striction effect of praseodymium 4f and Fe 3d spins, and hence the ferroelectricity only occurs below the praseodymium 4f spin ordering temperature [37]. In the present work, the polarization response stems from A-site ionic polarization and Fe^3+^↔Fe^2+^ hopping. In non-centrosymmetry, the octahedral distortion induced by Al^3+^ iron and quenching treatment contributes to praseodymium 4f ionic polarization. Non-magnetic ions with three filled 2p electron orbitals promote small electronic rearrangements in non-centrosymmetric distortion and increase the stress induced by square bond formation in B-sites. Also, the presence of a small number of Fe^2+^ ions boosts the Fe^3+^↔Fe^2+^ dipole hopping. 

**Figure 5:**
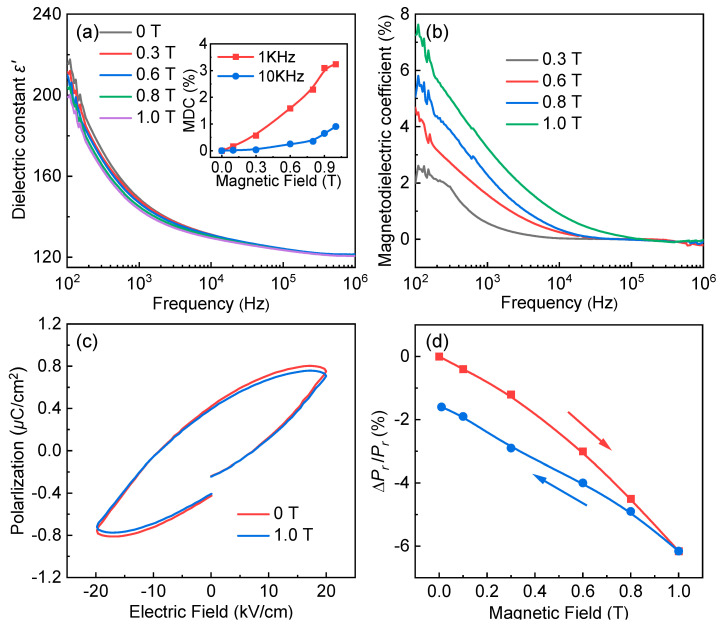
(**a**) Frequency dependence of (**a**) dielectric constant ε′ and (**b**) magnetodielectric coefficient (MDC) under different applied magnetic fields at room temperature, the inset of (**a**) shows the MDC dependence of the applied DC magnetic field; (**c**) P–E loops with and without magnetic field perpendicular to the thickness direction; (**d**) the estimated fractional change in Pr with H for the Pr_2_FeAlO_6_ compound.

Figure 5a depicts the frequency-dependent dielectric constant in various external magnetic fields at room temperature for Pr_2_FeAlO_6_. The dielectric constants significantly decrease with an increase in the applied DC magnetic field H, indicating the magnetodielectric effect. This effect is assessed by magnetodielectric coefficient MDC = [ε′(H)–ε′(0)]|/ε′(0), where ε′(0) and ε′(H) are measured under zero and nonzero magnetic fields, respectively. The maximum MDC value of 3.21% is achieved for the Pr_2_FeAlO_6_ at 1.0 T under the frequency of 1 kHz. In general, the MDC depends on the extrinsic effect of a combination of magnetoresistance and Maxwell-Wagner effect or an intrinsic effect of strain-induced coupling [38]. The measured magnetoresistance of (0.171 ± 0.04)% under the external magnetic field of 1.0 T show that there is no significant change in resistance for the Pr_2_FeAlO_6_, indicating that the magnetodielectric effect stems mainly from an intrinsic effect. Therefore, the observed ME coupling in the sample may be related to the strain coupling between the magnetic and ferroelectric domains. This can be confirmed by the changed remanent polarization shown in Figure 5c,d. The recorded ferroelectric hysteresis by applying a static magnetic field H (0~1.0 T) was done for the Pr_2_FeAlO_6_ compound, as shown in Figure 5c. One may clearly see that the P_r_ show an obvious reduction with an applied magnetic field. The change in P_r__,_ defined as ∆P_r_/P_r_ = [P_r_(H)−P_r_(0)]/P_r_(0), is plotted as a function of H in Figure 5d. ∆P_r_/P_r_ is negative and its magnitude is found to increase with increasing H. One also observes a hysteresis phenomenon. When H is decreased from 1.0 T to zero, the ∆P_r_/P_r_ data show a further decrease. The magnitude of ∆P_r_/P_r_ increases from 0 to 6.15% as H raises from 0 to 1.0 T. Due to the coupling between the magnetic and ferroelectric domains, an additional electric field would therefore be generated by the displacement of the ions (and possibly of the electronic clouds), and would be a consequence of the strain and in turn a secondary consequence of the stress (via the strain). This field orients the ferroelectric domains, giving rise to a high pinning of domains. Consequently, an obvious reduction and hysteresis phenomenon were observed. However, it is difficult to identify clearly how the magnetic field alters the polarization by current measurements, i.e., strain-mediated or ions interaction, or even both. One possibility is that the rearrangement of iron ions under the magnetic field may promote the disordered movement of magnetic moments of iron ions and subsequently the decreasing of the polarization. Therefore, future work is needed to understand the mechanism of magnetoelectric coupling. In addition, ∆P_s_/P_s_ ([P_s_(H)−P_s_(0)]/P_s_(0)) is defined by the changes P_s_ under the applied magnetic field H. The ∆P_s_/P_s_ is maximizing at 5.71 % with the value of 4.64 × 10^−5^ μC·cm^−^^2^·Oe^−^^1^. This value is comparable to that of Bi_0.5_Na_0.5_TiO_3_-La_0.67_Sr_0.33_MnO_3_ bulk composites (2.07 × 10^−5^ μC·cm^−^^2^·Oe^−^^1^) [39] and BaTiO_3_-NiFe_2_O_4_ core-shell composites (1.52 × 10^−5^ μC·cm^−^^2^·Oe^−^^1^) [40]. From the obtained results, we believe that the Pr_2_FeAlO_6_ sample will serve as a good multiferroic and have potential application in magnetoelectric or magnetodielectric devices.

## 4. Conclusions

In summary, a new double perovskite multiferroic Pr_2_FeAlO_6_ has been successfully synthesized using a sol-gel process followed by a quenching treatment. The material exhibits a coexistence of ferroelectric and ferromagnetic ordering above room temperature. Furthermore, the decreased dielectric constant and polarization under an external magnetic field evidence the ME coupling. Hence, Pr_2_FeAlO_6_ can be considered to be a room-temperature multiferroic compound for field-tunable applications.

## Data Availability

Data can be available upon request from the authors.

## Supplementary Materials

The following are available online at https://www.mdpi.com/article/10.3390/nano12173011/s1, Table S1 Rietveld refinement parameters of Pr_2_FeAlO_6_ by GSAS.
