# Supplementary material for: Multiferroic and Magnetodielectric Effects in Multiferroic Pr2FeAlO6 Double Perovskite
Source: Nanomaterials (Basel). 2022 Aug 30;12(17):3011. doi: 10.3390/nano12173011 (PMC9457962; doi:10.3390/nano12173011)
Supplement: Supplementary file 1 [file nanomaterials-12-03011-s001.zip › nanomaterials-1773110-supplementary.pdf]

Supplementary Materials

Multiferroic and Magnetodielectric Effects in Multiferroic Pr_2FeAlO_6 Double Perovskite

Sheng Liu [1], Feng Xiang [1,*], Yulan Cheng [1], Yajun Luo [1] and Jing Sun [2]

Table S1. Rietveld refinement parameters of Pr_2FeAlO_6 by GSAS.

Pr_2FeAlO_6	Parameters	Wyckoff	Element	*x*	*y*	*z*
Crystal structure	R3c					
Lattice a (Å)	5.511	6a	Pr	0.33	0.67	0.41
Lattice b (Å)	5.511					
Lattice c (Å)	13.067					
Volume (Å^3)	343.839	3a	Fe	0.67	0.33	0.31
R factors R_p (%)	3.55					
R factors w_{Rp} (%)	4.45					
R factors χ^2 (%)	2.39	3b	Al	0.33	0.67	0.16
U	1.113					
V	-1.427					
W	4.413	18f	O	0.68	0.90	0.07
Temperature factor	0.013					
